# Isolation of Mutants With Reduced Susceptibility to Piperaquine From a Mutator of the Rodent Malaria Parasite *Plasmodium berghei*

**DOI:** 10.3389/fcimb.2021.672691

**Published:** 2021-06-16

**Authors:** Mie Ikeda, Makoto Hirai, Shin-Ichiro Tachibana, Toshiyuki Mori, Toshihiro Mita

**Affiliations:** Department of Tropical Medicine and Parasitology, Faculty of Medicine, Juntendo University, Tokyo, Japan

**Keywords:** piperaquine resistance, mutator, CRT, fitness, *Plasmodium berghei*, mutants with reduced PPQ susceptibility 2

## Abstract

Elucidation of the mechanisms of drug resistance in malaria parasites is crucial for combatting the emergence and spread of resistant parasites, which can be achieved by tracing resistance-associated mutations and providing useful information for drug development. Previously, we produced a novel genetic tool, a *Plasmodium berghei* mutator (PbMut), whose base substitution rate is 36.5 times higher than that of wild-type parasites. Here, we report the isolation of a mutant with reduced susceptibility to piperaquine (PPQ) from PbMut under PPQ pressure by sequential nine-cycle screening and named it PbMut-PPQ-R-P9. The ED_50_ of PbMut-PPQ-R-P9 was 1.79 times higher than that of wild-type parasites, suggesting that its PPQ resistance is weak. In the 1^st^ screen, recrudescence occurred in the mice infected with PbMut but not in those infected with wild-type parasites, suggesting earlier emergence of PPQ-resistant parasites from PbMut. Whole-genome sequence analysis of PbMut-PPQ-R-P9 clones revealed that eight nonsynonymous mutations were conserved in all clones, including N331I in *PbCRT*, the gene encoding chloroquine resistance transporter (*CRT*). The PbCRT(N331I) mutation already existed in the parasite population after the 2^nd^ screen and was predominant in the population after the 8^th^ screen. An artificially inserted PbCRT(N331I) mutation gave rise to reduced PPQ susceptibility in genome-edited parasites (PbCRT-N331I). The PPQ susceptibility and growth rates of PbCRT-N331I parasites were significantly lower than those of PbMut-PPQ-R-P9, implying that additional mutations in the PbMut-PPQ-R9 parasites could compensate for the fitness cost of the PbCRT(N331I) mutation and contribute to reduced PPQ susceptibility. In summary, PbMut could serve as a novel genetic tool for predicting gene mutations responsible for drug resistance. Further study on PbMut-PPQ-R-P9 could identify genetic changes that compensate for fitness costs owing to drug resistance acquisition.

## Introduction

Malaria is one of the most life-threatening parasitic diseases worldwide. Its causative agents are *Plasmodium* spp., which are transmitted by anopheline mosquitoes. Among these, *P. falciparum* is the most malignant and threatens human life. It is responsible for 229 million clinical cases and 409,000 deaths, and its main victims are children under 5 years old living in sub-Saharan Africa (WHO and 2020, n.d). The obstacles for efficacious malaria control are the difficulty in development of effective vaccines and suppression of the emergence and spread of drug-resistant parasites. Regarding antimalarial drugs, the emergence of artemisinin-resistant malaria was confirmed in Cambodia in 2008 (Noedl et al., 2008), and the resistance has been spreading in the Greater Mekong subregion (Phyo et al., 2012; Kyaw et al., 2013). Since artemisinin is used in artemisinin-based combination therapy (ACT), treatment efficacy relies more heavily on the partner drug, which has induced the emergence of resistance to partner drugs, such as piperaquine (PPQ) (Amaratunga et al., 2016; Thanh et al., 2017). Moreover, the potential emergence of artemisinin-resistant *P. falciparum* parasites has been reported in Africa (Ikeda et al., 2018) and India (Das et al., 2018). The use of genetic markers is the most practical way to trace the emergence and spread of resistance. To identify genetic markers, a comparative genomics study between wild-type and drug-resistant parasites that emerged in the same area was performed. However, this approach is difficult and time-consuming because over 50,000 single-nucleotide polymorphisms (SNPs), corresponding to one SNP every 230 bp, are naturally detected in field isolates, e.g., from the border of China and Myanmar (Ye et al., 2019). In this context, isolation of drug-resistant parasites by *in vitro* evolution experiments could be simpler than the aforementioned approach. Moreover, isolation of drug-resistant parasites *in vitro* could predict mutations that will emerge in fields. For example, artemisinin-resistant parasites were isolated by *in vitro* under artemisinin pressure, and subsequent analysis identified a gene responsible for artemisinin resistance, *kelch13*. This attempt is still challenging because it took five years to isolate this mutant (Ariey et al., 2014). The reason for taking such a long time for the conventional *in vitro* evolution has been partly explained by the fact that the parasites amplify the genome surrounding the target genes through multiple steps, before the acquirement of mutations in the target genes (Guler et al., 2013). Therefore, it is anticipated that transgenic parasites producing spontaneous random nonsynonymous point mutations will produce drug-resistant parasites in a short time because several steps involved in genome amplification might be skipped. For this purpose, we previously generated a transgenic rodent malaria parasite, *P. berghei*, which possesses DNA polymerase δ with destructed proofreading activity (PbMut). The base substitution rate of PbMut is over 36.5-fold higher than that of wild-type parasites (Honma et al., 2014; Honma et al., 2016). Thus, PbMut could serve as a new tool for parasite drug resistance research (Hirai and Mita, 2015). In the present work, we attempted to isolate PPQ-resistant parasites from PbMut. As a result, we successfully isolated mutant clones exhibiting reduced susceptibility to PPQ and identified at least one SNP associated with this phenotype.

## Materials and Methods

### Animals and Parasites

Two mouse strains, ddY and BALB/c mice (female, 5 weeks old), were purchased from a supplier (Sankyo Labo Service Corp., Japan). ddY and BALB/c mice were used for piperaquine (PPQ)-resistant mutant screening and other experiments, respectively. The rodent malaria parasite *Plasmodium berghei* ANKA (clone 2.34) was used for the generation of a *P. berghei* mutator (PbMut) in our previous study (Honma et al., 2014). In brief, PbMut possesses mutations in the *DNA polymerase δ* gene destructing the proofreading activity in the corresponding gene product. In wild-type *P. berghei* (PbWT), endogenous *DNA polymerase δ* was replaced by the wild-type gene. Both transgenic parasites possess a pyrimethamine resistance gene as a marker. The parasites were maintained by weekly passaging through mice. After 91 weeks of passaging, the parasite population (PbMut-P91) was composed of various mutants that were used in PPQ-resistant mutant screening. The protocols for animal and recombinant DNA experiments were approved by the Experimental Animal Ethics Committee and Recombinant DNA Committee of the School of Medicine, Juntendo University, and the assigned numbers were no. 290017 and no. 25-115, respectively.

### Isolation of Piperaquine (PPQ)-Resistant Parasites From PbMut

**Figure 1:**
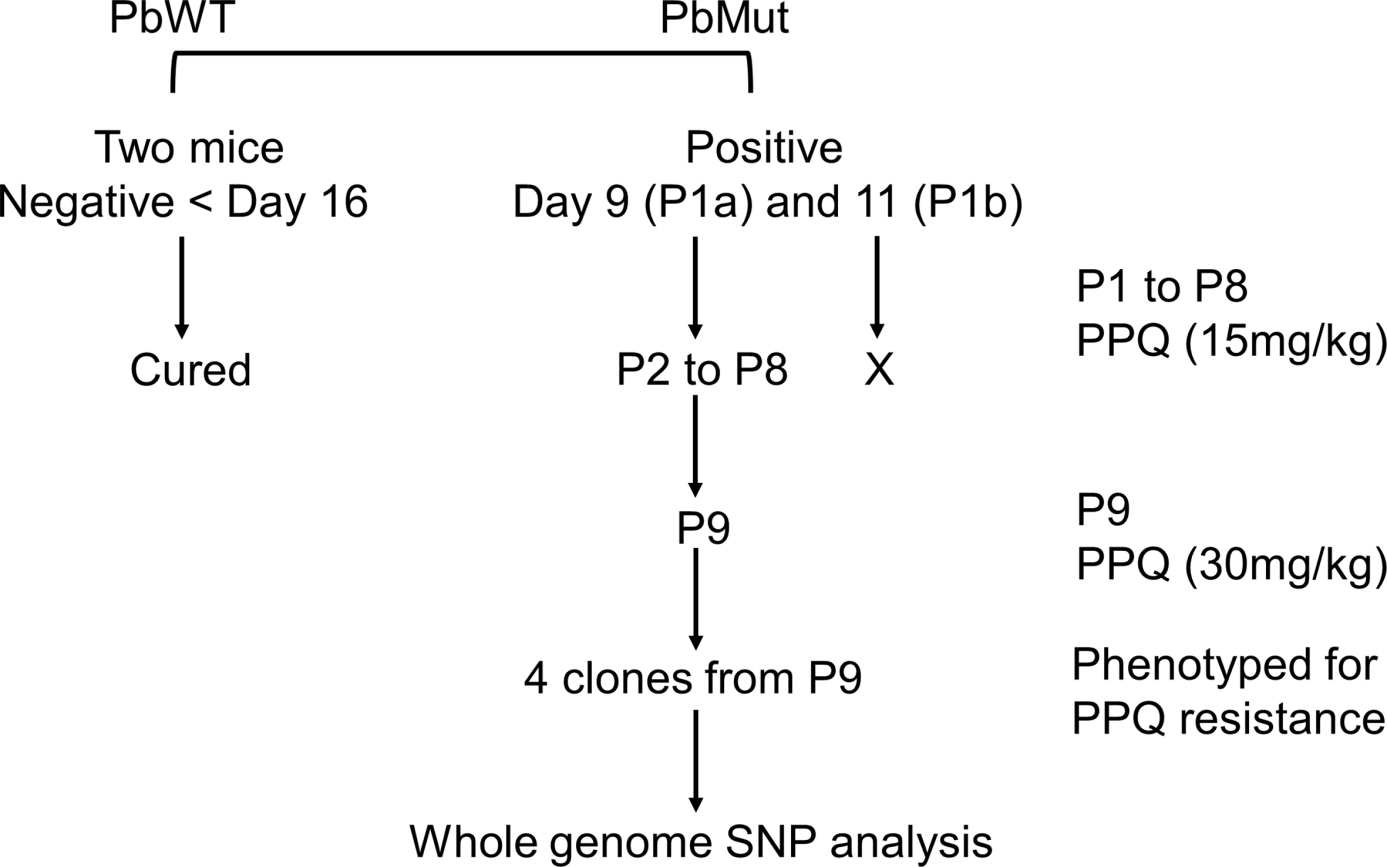
Flow chart of nine-cycle screening for PPQ-resistant parasites. At passage 1 (P1), recrudescence was not confirmed in PbWT-infected mice until day 16, while it was detected in both mice infected with PbMut (P1a and b). P1b failed to be passaged to the next mouse (indicated by a cross). P1a proceeded to sequential passages until P9. The four clones from P9 and one clone from the original PbMut (PbMut-P91 clone) were subjected to SNP analysis.

The procedure for PPQ-resistant parasite screening is described in **Figure 1**. On the 1^st^ screen, infected red blood cells (iRBCs, 1x10^6^) from the PbMut and PbWT populations were intravenously inoculated into two mice each. When parasitemia of the infected mice reached 1-2%, the mice infected with PbMut or PbWT were intraperitoneally administered PPQ tetraphosphate tetrahydrate (Sigma-Aldrich Co., USA) in Tween 80 (70%) and ethanol (30%) for four consecutive days. The drug administration start date was defined as day 0, and parasitemia was followed up until day 16. Parasitemia was quantified by counting over 2,000 erythrocytes on Giemsa-stained slides under a light microscope (1,000 x magnification). If infected erythrocytes were not detected by counting over 5,000 erythrocytes on day 16, we considered the mice to be cured. Recrudescence occurred in two mice carrying PbMut (PbMut-PPQ-R-P1a and b). These populations were passed to new mice, but the latter infection failed, and passaging was stopped thereafter. PbMut-PPQ-R-P1a was transferred to a mouse, which was treated with PPQ until passage 9 (P9), with PPQ doses of 15 and 30 mg/kg/day for P1 to P8 and P9, respectively. Four clones of PbMut-PPQ-R-P9 were isolated from the PbMut-PPQ-R-P9 population by limiting dilution.

To assess PPQ susceptibility, iRBCs (1x10^3^) of PbMut-PPQ-R-P9 clones or PbWT were injected intravenously into each mouse, which were administered PPQ (15 mg/kg/day) for 5 consecutive days. The parasitemia of PbMut-PPQ-R-P9 clones was monitored until day 11, while PbWT-infected mice were monitored until day 16.

Peters’s four-day suppressive test (Peters, 1965) was performed to compare the efficacy of PPQ between PbMut-PPQ-R-P9 clones and PbWT. In brief, iRBCs (1x10^6^) from three PbMut-PPQ-R-P9 clones or one PbWT clone were intravenously injected into five or fifteen BALB/c mice, respectively. Three mice infected with PbMut-PPQ-R-P9 or PbWT were then orally administered PPQ (2.5, 5, 7.5, or 10 mg/kg) at 4, 24, 48, and 72 hr post-infection. Three mice each were treated with 100 μl of Tween 80 (70%) and ethanol (30%) as a control. The parasitemia of all mice was quantified on day 4 post-infection. The 50% effective dose of PPQ (ED_50_), which is defined as the dose that suppresses 50% of parasitemia, was calculated by ICEstimator ver.1.2 online (http://www.antimalarial-icestimator.net/).

### Whole-Genome Sequencing and Single-Nucleotide Polymorphism (SNP) Analysis

In four PbMut-PPQ-R-P9 clones, and one PbMut-P91 clone which was obtained after 91 times serial passage through mice, genomic DNA was extracted from leukocyte-removed blood with a Plasmodipur filter (EuroProxima, The Netherlands) by using a QIAamp DNA Blood Mini Kit (QIAGEN, Germany) and sequenced with a HiSeq-2500 system (Illumina, USA). Analysis of genome-wide SNPs was conducted using CLC Genomics Workbench ver.11 (CLC-GW) (QIAGEN, Germany). Reads were mapped to the reference genome of *P. berghei* ANKA (PlasmoDB-49) with default parameters for detecting SNPs in samples. These steps for the selection of SNPs were performed using two variant detection tools (Basic Variant Detection and Fixed Ploidy Variant Detection) in CLC-GW. The PbMut-P91 SNPs were excluded from those of each PbMut-PPQ-R-P9 clone. The remaining nonsynonymous SNPs conserved in all clones were finally extracted as specific SNPs in PbMut-PPQ-R-P9 clones and were verified in all clones by Sanger sequencing. Multigene families were excluded from SNP analysis because of the high possibility of read mismapping.

### Monitoring the PbCRT-N331I Mutant in PbMut-PPQ-R Populations

To investigate the association of the PbCRT(N331I) mutation with PPQ susceptibility, the presence of PbCRT(N331I) was monitored in nine parasite populations (PbMut-PPQ-R-P1 to 9) obtained from each screen. For this, genomic DNA was extracted from each population and used as a template for PCR. A fragment covering PbCRT(N331) was amplified using a pair of primers, PbCRT-checkF and PbCRT-checkR. The PCR product was Sanger sequenced by using the PbCRT-SeqF primer. The primer sequences are shown in **Figure S2**.

### Generation of the PbCRT(N331I) Mutant by Genome Editing

The plasmid pYC for the CRISPR/Cas9 gene editing of *P. yoelii* was kindly gifted by Dr. Yuan (Zhang et al., 2014). To customize pYC for *P. berghei*, the U6 promoter of *P. yoelii* in pYC was excluded by inverse PCR using the primers A and B, which are designed in an outward direction from the *P. yoelii* U6 promoter region. The U6 promoter of *P. berghei* (PBANKA_1354380) was amplified with the primers C and D and ligated into the pYC PCR product by using an In-Fusion HD Cloning kit (TAKARA). The resultant plasmid, pBC, was used for *P. berghei* genome editing.

As donor DNA, a 1,343 bp fragment containing PbCRT(N331I) was synthesized (Genewiz, Japan) and ligated to the HindIII/AflII site in pBC by an In-Fusion HD Cloning kit. The candidate guide RNA (gRNA) was designed by the Eukaryotic Pathogen CRISPR Guide RNA Design Tool (http://grna.ctegd.uga.edu/). A double-stranded DNA coding for the candidate gRNA was ligated into the BsmBI site of pBC; therefore, the gRNA was transcribed under the *P. berghei* U6 promoter. The resultant plasmid, pPbCRT(N331I), was electroporated into wild-type *P. berghei*. Transfection and subsequent parasite cloning followed a previously described protocol (Janse et al., 2006). The mutation in the resultant PbCRT-N331I parasite clone was verified by Sanger sequencing. The structure of PbCRT(N331) plasmid and the sequence of PbCRT(N331I) parasite clone, and the donor DNA sequence are shown in **Figures S1A, B**, respectively. The sequences of the gRNAs and primers are shown in **Figure S2**.

### Comparison of PPQ Efficacy and Growth Rates Among PbMut-PPQ-R-P9, PbCRT-N331I, and PbWT Parasites

For the comparison of PPQ efficacy among PbMut-PPQ-R-P9, PbCRT-N331I, and PbWT, iRBCs (1x10^6^) from three clones of PbMut-PPQ-R-P9 or PbCRT-N331I were intravenously inoculated into three mice each. For PbWT, iRBCs (1x10^6^) were injected into nine mice. When the parasitemia reached 1-2%, PPQ (5, 15, and 30 mg/kg/day) was administered on day 0 for 5 consecutive days, and parasitemia was monitored until day 14. For PbWT-infected mice, parasitemia was followed up until day 16.

It is known that parasites acquire drug resistance at the expense of fitness (Rosenthal, 2013). Thus, the fitness of PbMut-PPQ-R-P9, PbCRT-N331I, and PbWT in each mouse was investigated. For this, iRBCs (1x10^6^) infected with each of the three parasite clones were inoculated into one mouse each. For PbWT, iRBCs were inoculated into three mice. Parasitemia was monitored from days 3 to 6 under drug-free conditions.

### Statistical Analysis

Comparisons between two or among three independent data groups were made by Student’s t-test or analysis of variance test (ANOVA) followed by Tukey’s multiple comparison test, respectively. Parasitemia is expressed as the mean ± standard deviation. P<0.05 was considered statistically significant.

## Results

### Isolation of PPQ-Resistant Parasites From PbMut

**Figure 2:**
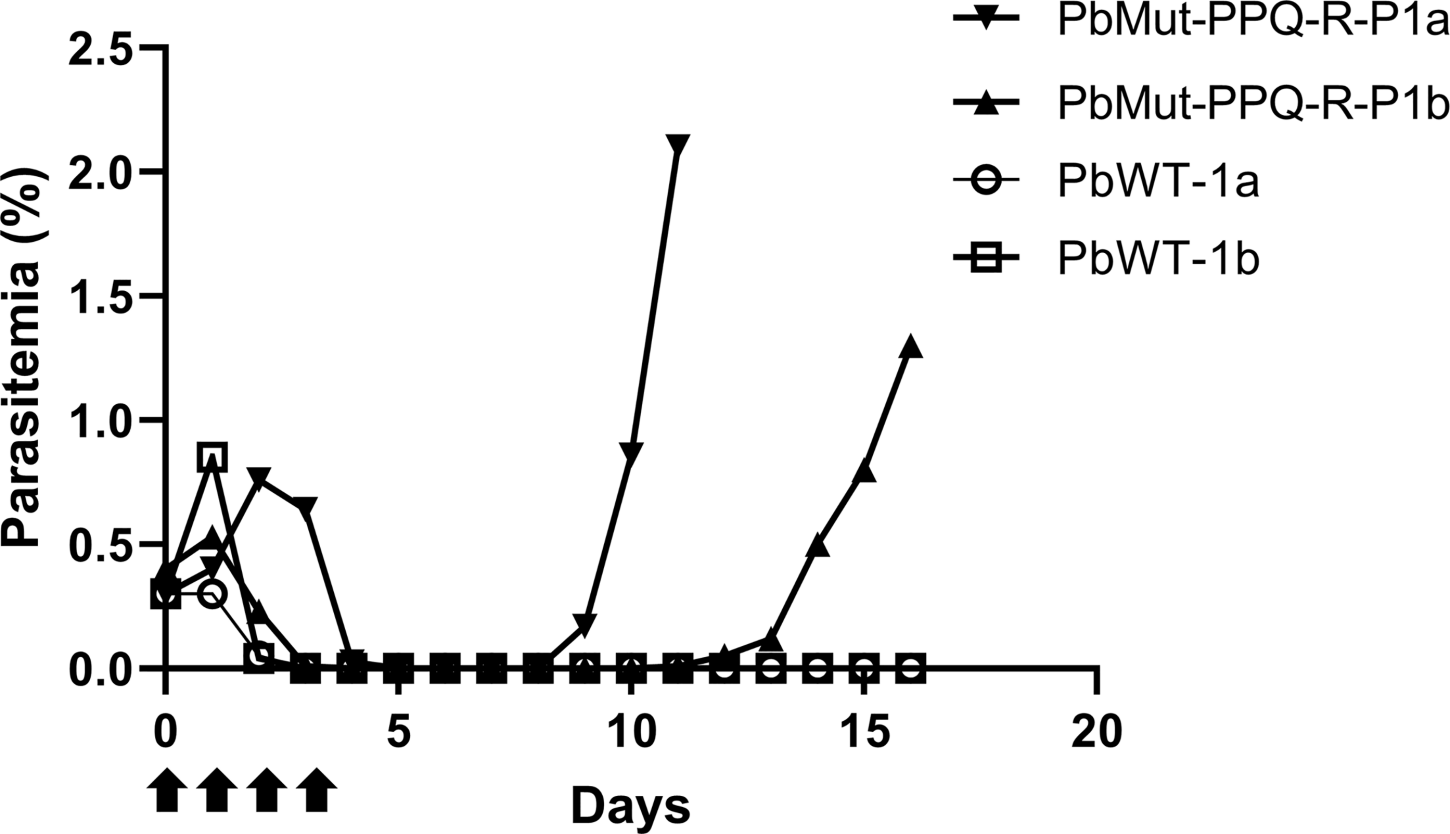
Screening for PPQ-resistant parasites from PbMut. The first screen for PPQ-resistant parasites. Two mice each were infected with PbMut (PbMut-PPQ-R-P1a; closed inverted triangle, and PbMut-PPQ-R-P1b; closed triangle) or PbWT (PbWT-P1a; open circle, and PbWT-P1b; open square). The mice were administered PPQ (15 mg/kg/day) for 4 days (arrows), and parasitemia was monitored.

**Figure 3:**
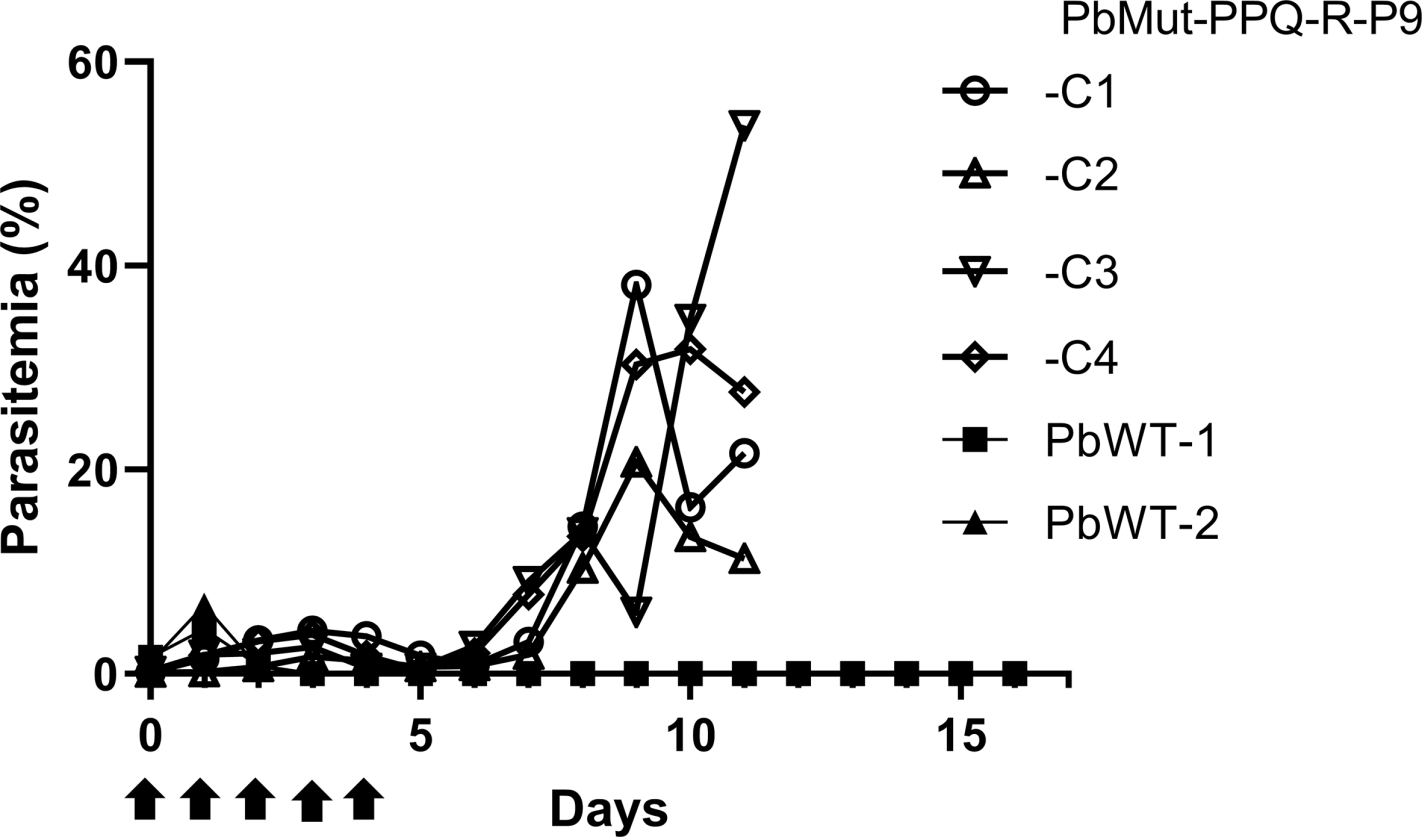
Assessment of PPQ susceptibility in PbMut-PPQ-R-P9-derived clones. Erythrocytes (1x10^3^) infected with two wild-type (PbWT-1 and PbWT-2, closed symbols) and four clones from PbMut-PPQ-R-P9 (open symbols) were transferred to each mouse. The mice were then treated with PPQ (15 mg/kg/day) for five consecutive days (arrows), and parasitemia was monitored.

To isolate PPQ-resistant parasites, two mice were infected with PbMut or PbWT parasites and were treated with PPQ (15 mg/kg/day, 4 days) on the 1^st^ screen. For PbWT-infected mice, infected erythrocytes were undetectable from day 3 until day 16. In contrast, PbMut-infected mice showed recrudescence on days 9 and 11 (PbMut-PPQ-R-P1a and -P1b) (**Figure 2** and **Table S1**). PbMut-PPQ-R-P1a and -P1b were each passaged to another mouse, but the latter failed to cause infection. Thus, only PbMut-PPQ-R-P1a proceeded to serial passaging until P9. After nine cycles of serial passaging, PbMut-PPQ-R-P9 parasites survived under 30 mg/kg PPQ treatment for five consecutive days (**Table S1**). Four clones derived from the PbMut-PPQ-R-P9 population were assessed for susceptibility to PPQ. As shown in **Figure 3**, all the clones survived under PPQ pressure (15 mg/kg/day, 5 days), but PbWT did not, suggesting that all the clones were less susceptible than PbWT to PPQ. A four-day suppressive test was performed to quantitatively assess PPQ susceptibility. The ED_50_ of PbMut-PPQ-R-P9 was 2.06 mg/kg, which is 1.79 times higher than that of PbWT (1.15 mg/kg). Such a slight rise in the ED_50_ for PbMut-PPQ-R-P9 suggests that its PPQ resistance is weak.

### Eight Nonsynonymous Mutations Shared Among the Four PbMut-PPQ-R-P9 Clones

To identify gene mutations responsible for the reduced PPQ susceptibility, we performed whole-genome SNP analysis in the four clones of PbMut-PPQ-R-P9 and one clone of PbMut-P91 as a control. Over three hundred redundant data points were obtained from each sample (**Table S2**). The numbers of nonsynonymous SNPs detected in PbMut-P91 and the four clones were 103, 113, 110, 123, and 117, respectively. SNPs of the four clones that were shared with PbMut-P91 were excluded, resulting in 20, 20, 31, and 25 SNPs for clones (**Table S3**). Of these, eight were conserved among all the clones (**Table 1**). Six of these eight nonsynonymous mutations were detected in the following genes*: exoribonuclease II* (SNP1), *tetratricopeptide repeat protein* (SNP3), *rhomboid protease ROM8* (SNP4) (Lin et al., 2013), *AP-2 complex subunit α* (SNP5), *chloroquine resistance transporter* (*CRT*) (SNP6) (Fidock et al., 2000; Martin et al., 2009; Juge et al., 2015) and *AAP4* (SNP8). Mutations in *CRT* of *P. falciparum* (*PfCRT*) have been suggested to be associated with resistance against several antimalarials, including PPQ (Agrawal, 2017; Ross et al., 2018). To investigate the association of PbCRT(N331I) with reduced PPQ susceptibility, the PbCRT(N331I) mutation in the nine parasite populations was monitored. This mutation was detected in the P2 population, and the wild-type allele was replaced by this mutation in the P8 and P9 populations (**Figure S3**), suggesting that this mutation might be selected by PPQ pressure.

**Table 1:** Eight SNPs conserved in 4 clones of PbMut-PPQ-R-P9.

SNP site	Chr.	Position	Gene ID	Gene description	Amino acid change	Expression	KO
SNP1	4	552412	PBANKA_041530	exoribonuclease II, putative	K91N	A>S	×
SNP2	7	197615	PBANKA_070480	conserved Plasmodium protein	K349Q	A<S	?
SNP3	8	1048775	PBANKA_082730	tetratricopeptide repeat protein	K864N	A<S	?
SNP4	10	1225025	PBANKA_103130	rhomboid protease ROM8	P435S	A/S	×
SNP5	11	611718	PBANKA_111670	AP-2 complex subunit alpha, putative	L1128I	A/S	?
SNP6	12	722508	PBANKA_121950	chloroquine resistance transporter	N331I	A>S	×
SNP7	13	1765633	PBANKA_134480	conserved Plasmodium protein	R54K	A>S	?
SNP8	14	631065	PBANKA_141720	protein AAP4, putative	I773L	A<S	?

The SNPs are listed in order of chromosome number.

A, asexual; S, sexual stages; A>S, A<S and A/S represent the relative abundance of transcription between these two stages x and ○ represent failed and success for knockout.? is unknown. All data are referred from PlasmoDB.

### Comparison of PPQ Susceptibility and Growth Rates Among PbMut-PPQ-R-P9, PbCRT-N331I, and PbWT Parasites

**Figure 4:**
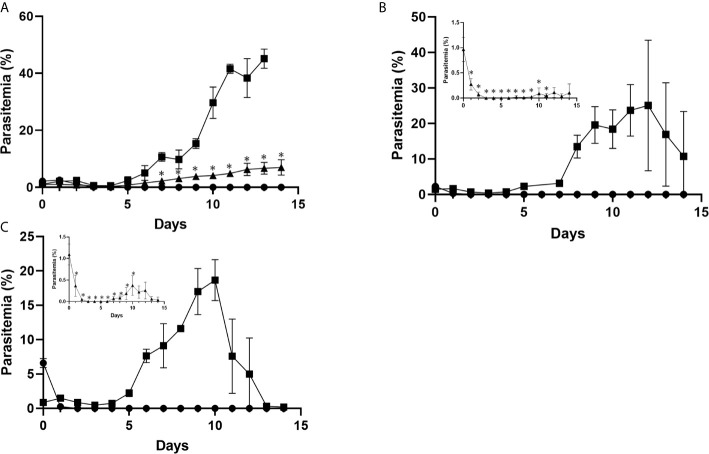
Comparison of PPQ susceptibility among PbCRT-N331I, PbMut-PPQ-R-P9, and PbWT parasites. Three clones of PbMut-PPQ-R-P9 (closed square) or PbCRT-N331I (closed triangle) were intravenously inoculated into three BALB/c mice each. PbWT parasites (closed circle) were injected into nine mice. Three mice in each group were then treated with PPQ at doses of 5, 15, and 30 mg/kg/day **(A–C)** for five consecutive days starting on day 0. The parasitemia of PbCRT-N331I is shown in the insets of **(B, C)** because of low parasitemia. As shown in **(A)**, the parasitemia of PbMut-PPQ-R-P9 on day 14 was not determined because the mice died due to high parasitemia. The bar represents the standard deviation. The asterisks indicate that the parasitemia of PbCRT-N331I was significantly different from that of PbMut-PPQ-R-P9 (p<0.05).

Transgenic PbCRT-N331I and PbMut-PPQ-R-P9, as well as PbWT parasites as controls, were inoculated into mice that were exposed to PPQ. Both PbMut-PPQ-R-P9 and PbCRT-N331I survived under PPQ pressure, but PbWT did not (**Figure 4**). Notably, the parasitemia of PbCRT-N331I was less than that of PbMut-PPQ-R-P9 (**Figure 4**). This result suggests that PbMut-PPQ-R-P9 could be less susceptible than PbCRT-N331I to PPQ. In addition, it is interesting that no suppressive effect of a higher PPQ dose on parasitemia was detected for PbCRT-N331I (insets, **Figures 4B, C**). For PbMut-PPQ-R-P9, parasitemia continuously increased until mouse death on day 15 at a PPQ dose of 5 mg/kg/day (**Figure 4A**). At PPQ doses of 15 and 30 mg/kg/day, parasitemia peaked on day 12 (**Figure 4B**) and day 10 (**Figure 4C**) and then declined. The decrease in parasitemia might be due to host immunity and/or the accumulative effect of PPQ pressure, which could suppress parasite growth later in the follow-up period.

**Figure 5:**
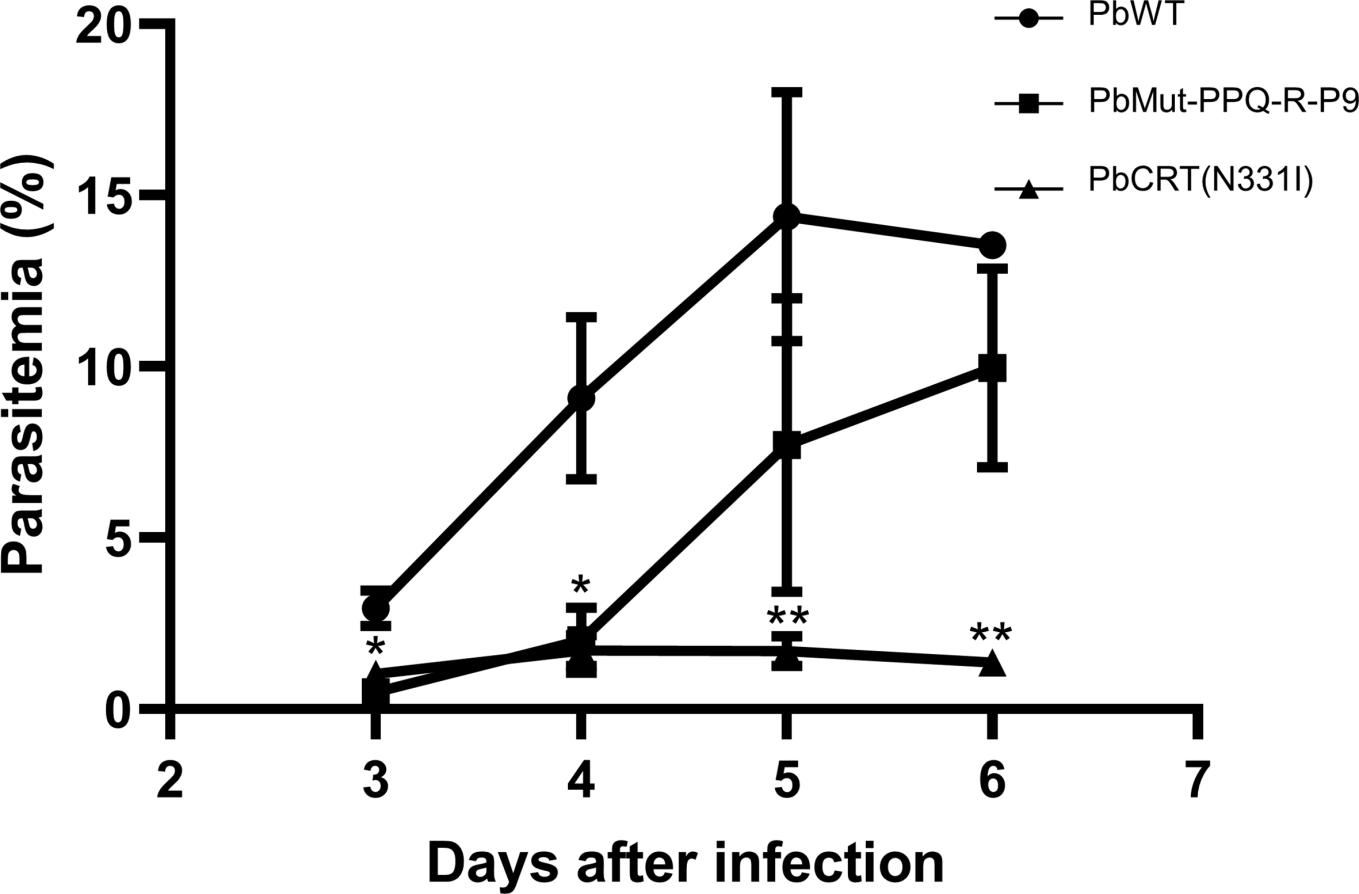
Comparison of growth rates among PbCRT-N331I, PbMut-PPQ-R-P9 and PbWT parasites. Three clones of PbCRT-N331I (closed triangle) and PbMut-PPQ-R-P9 (closed square) were inoculated into one mouse each. PbWT (closed circle) was inoculated into three mice. Parasitemia was monitored from days 3 to 6 after infection. On days 3 and 4, the parasitemia of PbCRT-N331I and PbMut-PPQ-R-P9 was significantly lower than that of PbWT (p <0.05, indicated by a single asterisk). On days 5 and 6, the parasitemia of the PbCRT-N331I parasite was significantly lower than that of PbMut-PPQ-R-P9 and PbWT (p <0.05, indicated by double asterisks). The bar represents the standard deviation.

To investigate the possibility that the PbCRT(N331I) mutation imposes fitness costs on the parasite, the growth rates of PbMut-PPQ-R-P9 and PbCRT(N331I), as well as of PbWT, in mice were compared under drug-free conditions. The parasitemia of PbCRT-N331I and PbMut-PPQ-R-P9 was significantly less than that of PbWT on days 3 and 4 post-infection (* p<0.05, **Figure 5**). On days 5 and 6, the parasitemia of PbCRT-N331I was less than that of PbMut-PPQ-R-P9 (** p<0.05, **Figure 5**). This result demonstrates that the PbCRT(N331I) mutation partially impaired parasite growth in mice. This result also suggests that some genetic factors existing in PbMut-PPQ-R-P9 could compensate for the fitness cost imposed by the PbCRT(N331I) mutation.

## Discussion

In this study, we isolated PbMut-PPQ-R-P9 from PbMut after nine rounds of passaging with PPQ administration. The ED_50_ of PbMut-PPQ-R-P9 was 1.79 times higher than that of PbWT. Despite the weak PPQ resistance of PbMut-PPQ-R-P9, we successfully identified eight nonsynonymous SNPs that were conserved in all clones of PbMut-PPQ-R-P9 (**Table 1**). Four SNPs (SNP2, 3, 7, and 8) were detected in genes that are exclusively expressed at sexual stages and/or encode hypothetical proteins with no remarkable functional domain (PlasmoDB; https://plasmodb.org/plasmo/). SNP4 is in the rhomboid protein family gene (ROM8), which plays critical roles in host cell invasion in apicomplexan parasites (Lin et al., 2013) and is unlikely to be related to PPQ susceptibility. SNP1 is in the gene coding for PbRNase II. In *P. falciparum*, an ortholog of PbRNase II functions in posttranscriptional silencing of *upsA*, a *var* subgroup gene (Zhang et al., 2014) and has not been reported to have an association with PPQ resistance. Finally, either of the two remaining SNPs may be responsible for PPQ susceptibility, which is in *PbCRT* (SNP6) and *AP2-α* (SNP5).

We focused on PbCRT(N331I) because CRT is a well-known transporter on the food vacuole, a principal site of quinoline antimalarials, such as PPQ (Roepe, 2011; Ecker et al., 2012). To investigate the association of PbCRT(N331I) with PPQ susceptibility, we generated PbCRT-N331I parasites and confirmed that PbCRT-N331I exhibited reduced PPQ susceptibility (**Figure 4**). PbCRT(N331) corresponds to N330 in PfCRT (Kim et al., 2019). There is no evidence for the occurrence of PfCRT(N330I) in 3,488 *P. falciparum* genomes from 23 countries (Project, M. falciparum, 2016). Instead, a decrease in PPQ susceptibility or resistance has been significantly associated with particular mutations in *PfCRT*, including T93S, H97Y, F145I, I218F, M343L, and G353V in Southeast Asia and C350R in South America (Pelleau et al., 2015; Ross et al., 2018; Dhingra et al., 2019; Hamilton et al., 2019). Direct evidence of these associations from transfection studies using the Dd2 clone has been reported for all the above Southeast Asian mutations; T93S and I218F (Dhingra et al., 2019); H97Y, F145I, M343L, and G353V (Ross et al., 2018) and C350R using the 7G8 clone (Pelleau et al., 2015). Accumulating evidence has revealed that all these mutations reside within a negatively charged cavity in PfCRT where PPQ binds (Kim et al., 2019) and that these mutations change in the electrostatic potential to promote the escape of protonated PPQ from the food vacuole (Coppée et al., 2020), thereby contributing to PPQ resistance/susceptibility. According to the structural model of PfCRT (Kim et al., 2019), PfCRT(N330) appears to localize to the cavity (Protein Data Bank accession no. 6UKJ) (Kim et al., 2019). Thus, it is likely that PbCRT(N331) may also be located in the cavity and contribute to reduced PPQ susceptibility.

The growth of PbCRT-N331I parasites was much slower than that of wild-type parasites (**Figure 5**), suggesting that PbCRT(N331I) may impose a fitness cost. Relatedly, PfCRT(N330I) has not been detected in natural parasite populations (Project, M. falciparum, 2016). It is therefore likely that PfCRT-N330I parasites may not persist in natural populations because of the possible fitness cost of PfCRT(N330I). A supportive example was seen in the PPQ-resistant PfCRT-C101F mutant generated from *in vitro* culture (Eastman et al., 2011). PfCRT-C101F exhibited a much slower replication rate than wild-type parasites (Dhingra et al., 2017) and has not been detected in field samples (Project, M. falciparum, 2016). The absence of PfCRT-N330I in natural parasite populations does not mean that PbMut cannot reproduce PPQ-resistant mechanisms occurring in field parasites. Rather, PbMut could highlight the importance of CRT, especially mutations in the cavity of CRT, for PPQ resistance/susceptibility.

*AP2-α* (SNP5), a candidate SNP for reduced PPQ susceptibility, is a heterotetrameric complex that is involved in endocytosis (Mettlen et al., 2018). In *P. falciparum*, AP2 is composed of the α, β, μ, and σ subunits (Henrici et al., 2020), particular mutations in which were shown to be associated with drug resistance. The S160N mutation in *AP2-µ* enhanced parasite survival following ACT in Kenya (Henriques et al., 2014). A transfection study revealed that this mutation slightly decreased the *in vitro* sensitivity to quinine (Henriques et al., 2015). AP2-µ has been reported to interact with kelch13 and play roles in hemoglobin internalization and degradation, which regulates heme-dependent artemisinin activation (Yang et al., 2019; Birnbaum et al., 2020). Since the antimalarial action of PPQ is thought to inhibit the detoxification of toxic heme in food vacuoles (Blasco et al., 2017), we speculate that our identified AP2-α mutation might reduce PPQ potency *via* a decrease in hemoglobin uptake.

It has been proposed that copy number variation of *plasmepsin II/III* (*PfPmII/III*) is associated with PPQ susceptibility/resistance in *P. falciparum* (Amato et al., 2017; Witkowski et al., 2017; Bopp et al., 2018). In our study, however, *PbPmIV* (PBANKA_1034400), an ortholog of *PfPmII*, was encoded as a single copy in PbMut-PPQ-R-P9 (data not shown). In support of this finding, the introduction of several PfCRT mutations (H97Y, F145I, M343L, or G353V) into Dd2 gave rise to a PPQ-resistant phenotype without multicopying PfPmII/III (Ross et al., 2018). Overexpression of *PfPmII/III* in 3D7 did not confer PPQ resistance to the parasites (Loesbanluechai et al., 2018). These data suggest dispensable roles of multicopied *PfPmII/III* for PPQ resistance. Besides, PbMut-PPQ-R-P9 has weak PPQ resistance because its ED_50_ is low (2.06 mg/kg) and is much lower than that (168.08 mg/kg) of PPQ-resistant parasites previously isolated by another group (Kiboi et al., 2009). Thus, PbMut is in the middle of an evolutionary process for acquiring potent PPQ resistance. Whether copy number elevation of *PbPmIV* occurs in PPQ-resistant PbMut parasites may answer the question of its association with PPQ resistance.

Overall, we provide evidence showing that PbMut could serve as a novel forward genetic tool. We previously showed that PbMut populations accumulated many mutations with an increase in the number of serial passages and that each clone had few overlapping mutations (Honma et al., 2016). Therefore, in this experiment, we used PbMut-P91, a high passage number PbMut library, to screen as many mutants as possible for quick isolation of mutants with reduced PPQ susceptibility. Indeed, recrudescence occurred in the mice infected with PbMut but not PbWT at the 1^st^ screen (**Figure 2** and **Table S1**). PbCRT(N331I) already emerged in the parasite populations after the 2^nd^ screen and then outcompeted wild-type parasites in the population after the 8^th^ screen (**Figure S3**). Such rapid emergence of PbCRT(N331I) suggests that it may preexist in the PbMut-P91 population below the detectable limit. It is assumed that a drug-resistant phenotype imposes fitness costs that could be partially compensated by background gene mutations. Mutations such as those in ferredoxin, apicoplast ribosomal protein S10, multidrug resistance 2, and CRT are thought to create a genetic foundation suitable for kelch13 mutations in parasites from Southeast Asia (Miotto et al., 2015). In our study, the growth rate of PbMut-PPQ-R-P9 was higher than that of PbCRT-N331I (**Figure 5**), suggesting that background mutations might exist in PbMut-PPQ-R-P9 that compensate for fitness costs owing to PbCRT(N331I) acquisition. Currently, comparative SNP analysis among parasite populations obtained after each screen (PbMut-PPQ-R-P1 to P9) is ongoing. This study direction could provide clues for understanding genetic mechanisms in the fitness elevation of drug-resistant parasites, which is one of the critical factors for the spread and fixation of drug-resistant parasites in the field.

## Data Availability Statement

The datasets presented in this study can be found in online repositories. DDBJ repository (https://www.ddbj.nig.ac.jp/index-e.html), accession number: DRA010243.

## Ethics Statement

The animal study was reviewed and approved by Experimental Animal Ethics Committee of the School of Medicine, Juntendo University, and the assigned numbers were no. 290017.

## Author Contributions

MI and MH performed the experiments. MH, SI-T, TMo and TMi analyzed the data. MH and TMi wrote the manuscript and supervised the work. All authors contributed to the article and approved the submitted version.

## Funding

This work was supported by JSPS KAKENHI for Scientific Research (B) and (C) to TMi. (17H04074) and MH (18K07095), respectively. This work was also supported by the Research Program on Emerging and Re-emerging Infectious Diseases (21fk0108138s0402) from the Japan Agency for Medical Research and Development, AMED, to MH.

## Conflict of Interest

The authors declare that the research was conducted in the absence of any commercial or financial relationships that could be construed as a potential conflict of interest.
