# Supplementary figures and images for: Isolation of Mutants With Reduced Susceptibility to Piperaquine From a Mutator of the Rodent Malaria Parasite Plasmodium berghei
Source: Front Cell Infect Microbiol. 2021 Jun 16;11:672691. doi: 10.3389/fcimb.2021.672691 (PMC8242943; doi:10.3389/fcimb.2021.672691)

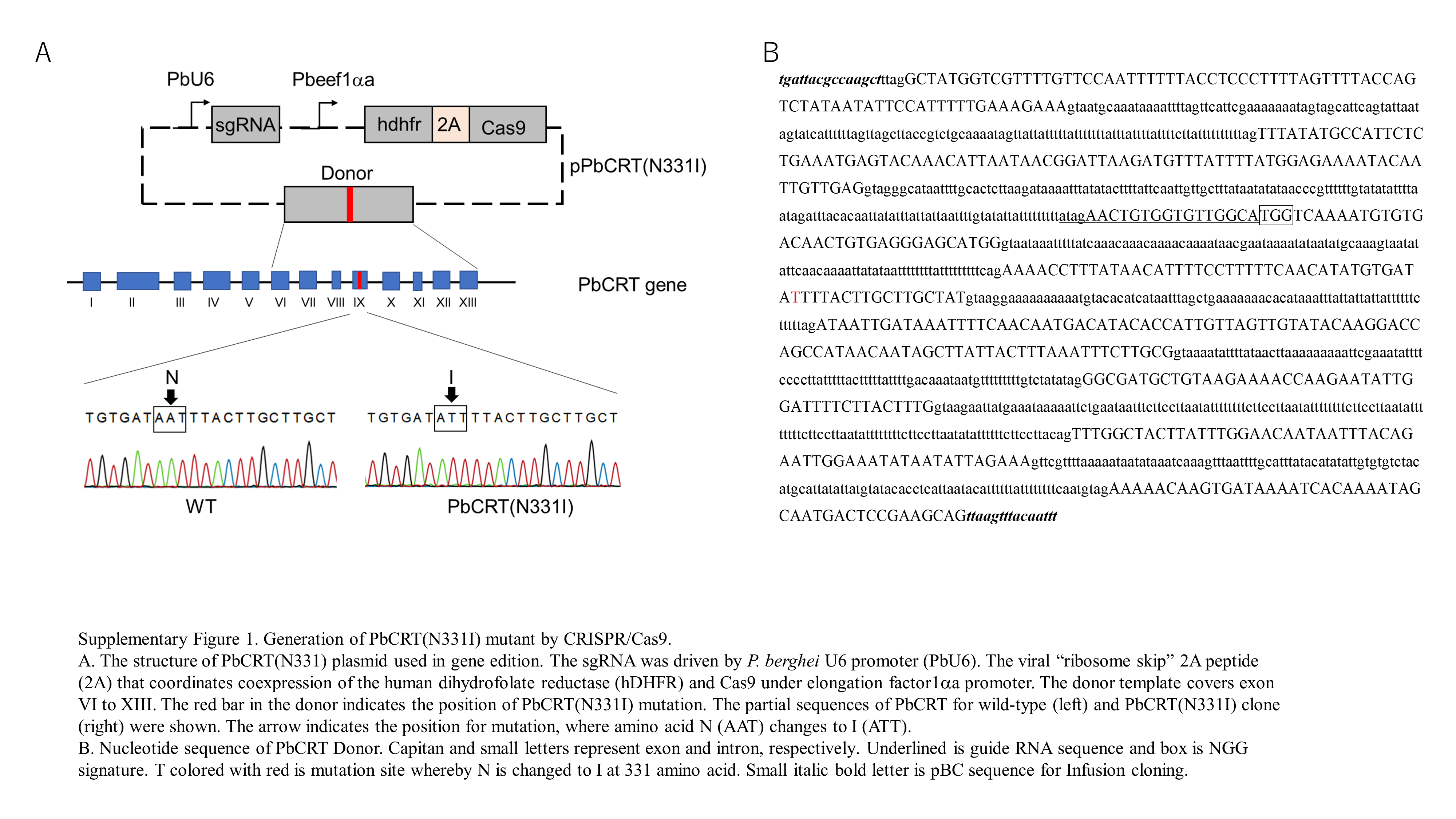

Supplement: Supplementary file 1 [file Image_1.tif]
