# Supplementary material for: Isolation of Mutants With Reduced Susceptibility to Piperaquine From a Mutator of the Rodent Malaria Parasite Plasmodium berghei
Source: Front Cell Infect Microbiol. 2021 Jun 16;11:672691. doi: 10.3389/fcimb.2021.672691 (PMC8242943; doi:10.3389/fcimb.2021.672691)
Supplement: Supplementary file 2 [file Presentation_1.pdf]

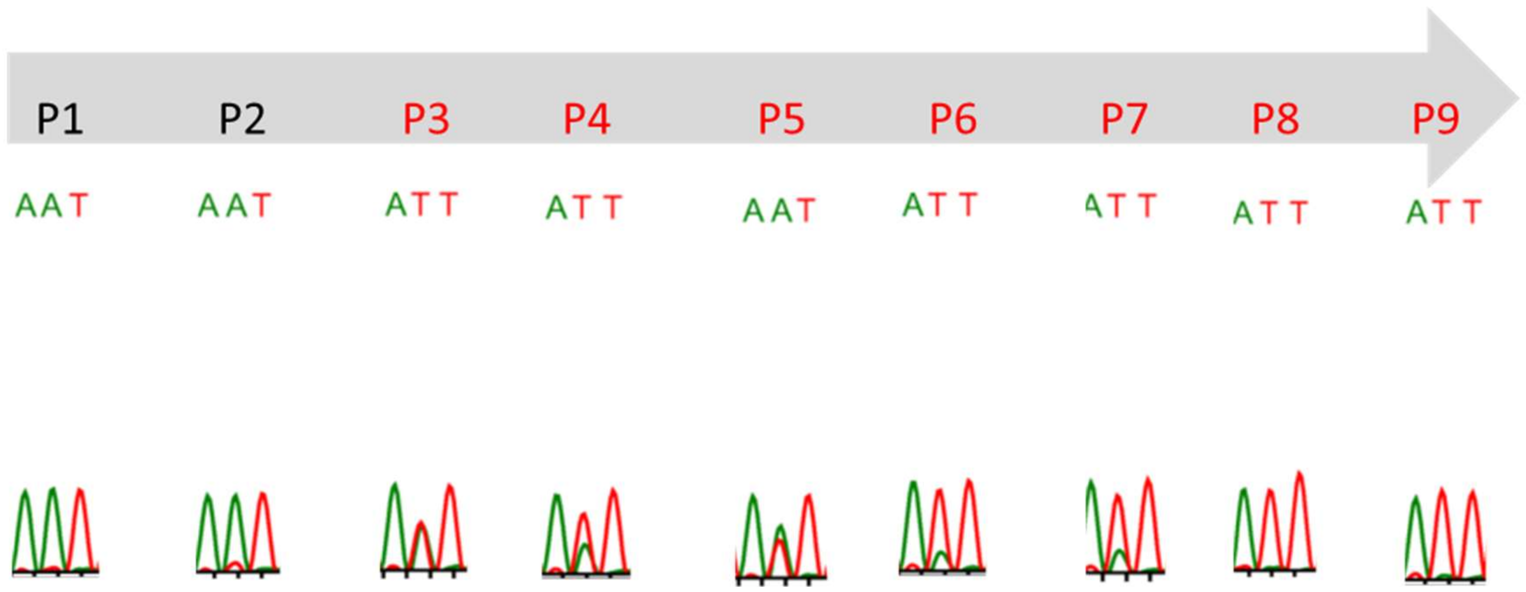

Fig. S3. The presence of PbCRT(N331I) mutation in the parasite populations.
The nine parasite populations (P1 to 9) were obtained after each screening. *PbCRT* gene encompassing N331I was amplified using genomic DNA of each populations. The middle A (wild-type) was replaced by T (mutation) in P8, suggesting high prevalence of PbCRT(N331I) mutation in P8 and P9 population under PPQ pressure.
